# Abnormal Neural Responses to Social Exclusion in Schizophrenia

**DOI:** 10.1371/journal.pone.0042608

**Published:** 2012-08-16

**Authors:** Victoria B. Gradin, Gordon Waiter, Poornima Kumar, Catriona Stickle, Maarten Milders, Keith Matthews, Ian Reid, Jeremy Hall, J. Douglas Steele

**Affiliations:** 1 Medical Research Institute, University of Dundee, Dundee, United Kingdom; 2 Biomedical Imaging Center, University of Aberdeen, Aberdeen, United Kingdom; 3 Department of Psychiatry, University of Oxford, Oxford, United Kingdom; 4 Institute of Mental Health, University of Aberdeen, Aberdeen, United Kingdom; 5 Department of Psychology, University of Aberdeen, Aberdeen, United Kingdom; 6 Division of Psychiatry, University of Edinburgh, Edinburgh, United Kingdom; George Mason University/Krasnow Institute for Advanced Study, United States of America

## Abstract

Social exclusion is an influential concept in politics, mental health and social psychology. Studies on healthy subjects have implicated the medial prefrontal cortex (mPFC), a region involved in emotional and social information processing, in neural responses to social exclusion. Impairments in social interactions are common in schizophrenia and are associated with reduced quality of life. Core symptoms such as delusions usually have a social content. However little is known about the neural underpinnings of social abnormalities. The aim of this study was to investigate the neural substrates of social exclusion in schizophrenia. Patients with schizophrenia and healthy controls underwent fMRI while participating in a popular social exclusion paradigm. This task involves passing a ‘ball’ between the participant and two cartoon representations of other subjects. The extent of social exclusion (ball not being passed to the participant) was parametrically varied throughout the task. Replicating previous findings, increasing social exclusion activated the mPFC in controls. In contrast, patients with schizophrenia failed to modulate mPFC responses with increasing exclusion. Furthermore, the blunted response to exclusion correlated with increased severity of positive symptoms. These data support the hypothesis that the neural response to social exclusion differs in schizophrenia, highlighting the mPFC as a potential substrate of impaired social interactions.

## Introduction

Difficulties in social interactions are a core feature of schizophrenia. This often adversely affects relationships, work functioning and independent living [1], [2]. Positive symptoms such as delusions and hallucinations usually have a prominent social content whilst negative symptoms include deficits in motivation, affect and social skills [3]. A review and meta-analysis of emotion perception studies [4] concluded that impairments in the ability to infer emotions is a robust finding in schizophrenia. Similarly, a meta-analysis of Theory of Mind studies (ToM; ability to make inferences about self or other mental states) [5] reported highly significant mentalising impairments in schizophrenia. Importantly, these impairments in social cognition have been linked to poor clinical outcome [2], [6]. Currently there are no effective *specific* treatments, highlighting the importance of improving understanding of the neural mechanisms underlying these abnormalities.

To date, few fMRI studies (see review [7]) have investigated the neural correlates of social impairments in schizophrenia. One of the brain regions most consistently implicated is the medial Prefrontal Cortex (mPFC). In healthy subjects, the mPFC has been consistently reported to be activated in ToM tasks [8] and it is frequently activated in emotion perception and induction studies [7], [9]. This highlights the importance of this brain region for emotional and social information processing.

In many social contexts it is important for humans to be socially accepted and not excluded. Victims of ostracism usually react with psychological discomfort (e.g. low mood and anxiety) and it has been argued that a number of robust social psychology phenomena can be explained by the notion that healthy individuals typically fear exclusion, rejection and being ignored [10]. In recent years fMRI studies have started to investigate the neural substrates of social exclusion in healthy subjects [11]–[15]. The fMRI paradigm that has been most used in studies of social exclusion is the ‘Cyberball’ task [10]. In this task participants play a ball-passing game with two animated cartoon figures whose actions are pre-programmed, such that the ‘real’ participant is at different times included and excluded. In a recent study [13] that used a carefully designed version of Cyberball, mPFC activation in response to social exclusion was found, which included the ventral anterior cingulate cortex (vACC), subgenual ACC and orbitofrontal cortex (OFC). The authors interpreted mPFC activation as related to processes of self-evaluation, inferences about other's thoughts and monitoring of social exchange outcomes to guide flexible behaviour, since these processes have been linked to mPFC functioning and are plausibly triggered by social exclusion [8], [13]. Consistent with Sebastian and colleagues report, two recent Cyberball studies have also reported mPFC/vACC activation in response to social exclusion [14], [15]. Other regions that have also been reported to exhibit responses to social exclusion are the dorsal anterior cingulate, amygdala, hippocampus, periaqueductal gray, anterior insula and the ventrolateral PFC [11], [12].

A neuroimaging study of schizophrenia using the Cyberball paradigm is of interest for several reasons. First, as above, such patients often show difficulties in social interactions [2]. To date though, most social studies of schizophrenia have used emotion perception or ToM paradigms that involve social interpretations but not interactions, with only a few studies of any type investigating the neural substrates of abnormalities [7]. The Cyberball paradigm aims to recreate in the scanner an experience involving both social interpretations and social interactions. Second, from the perspective of clinical research, this paradigm has also the advantage that it has been used in a number of studies on healthy subjects, which facilitates interpretation of findings in a clinical context. Third, studying social exclusion in schizophrenia is valuable since such patients may be especially affected by ostracism [16]. This is because: difficulties in social interactions may result in exclusion from work and relationships; insufficient income may prevent patients from participating in social activities [17]; people may feel more comfortable distancing themselves from people with schizophrenia [18].

In the present study we investigated the neural responses to social exclusion in schizophrenia using fMRI and a version of the Cyberball paradigm. The rostral and ventral mPFC was of particular interest as this region has been consistently linked to emotional and social information processing [8], reported to activate during social exclusion [13]–[15] and reported to exhibit abnormal activation in other social cognition studies of schizophrenia [7]. Our main hypothesis was that schizophrenia would be associated with mPFC abnormalities in response to imposed social exclusion. Additionally, we aimed to examine whether core illness severity ratings could explain the variance in mPFC activity in any regions identified as abnormal between patient and control groups.

## Materials and Methods

### Participants

The study was approved by the Grampian Local Research Ethics Committee. Potential patients and controls were given the Ethics approved Information Sheet and encouraged to discuss the study with others and take several days to decide on any questions. After a few days subjects were invited to meet with one of the researchers (JDS) and a discussion determined whether they understood the nature of the study and if they had further questions which were then answered. If they understood the task, wished to participate and met the inclusion criteria they were recruited. Written informed consent was obtained from all participants. The consent form was signed by the participants themselves. Data was acquired from two groups of subjects: a group of 15 patients with DSM IV schizophrenia and a group of 20 healthy controls. Exclusion criteria were any neurological disorder, claustrophobia, or other DSM IV Axis I or II diagnosis.

Patients were recruited via NHS Consultant Psychiatrists from their Community Mental Health Teams (CMHTs). All were outpatients in long term follow up at the time of scanning with stable chronic symptomatic illness despite on-going antipsychotic treatment. No advertisements were used for recruitment. All patients had been diagnosed with schizophrenia by Consultant Psychiatrists at least 2 years prior to recruitment and in many cases had been diagnosed decades earlier. For patients with a primary diagnosis of schizophrenia in long term NHS follow up, there is often significant comorbidity, particularly mood disorder and substance misuse. No patients satisfied criteria for a depressive illness at the time of scanning and no patients had a significant problem with substance misuse. With the exception of comorbidity, patients were typical of those seen in NHS outpatients.

Four control and two patient data sets were excluded because of structural brain abnormalities, failure to understand the task or scanner hardware failure. Sixteen controls and thirteen schizophrenia patients were finally included in the analysis. The two groups did not differ on a between group t-test with respect to age and National Adult Reading Test estimated pre-morbid IQ (Nelson and Wilson, 1991). Given the smaller proportion of females in the schizophrenia group than in the control group, gender was used as a covariate for the behavioural and image analyses. Details of subjects included in the analysis are presented in Table 1. Table S1 describes patient's antipsychotic medication at the time of the study.

**Table 1:** Patient and control details.

	Controls	Schizophrenia	Significance
Age (years)	40.87±11.72	41.23±11.78	p = 0.936
Gender (M/F)	7/9	11/2	
NART	113.57±8.30	106.55±11.92	p = 0.096
BDI	3.31±2.96	17.43±12.88	p = 0.002*
SP	30.86±10.97	45.07±12.18	p = 0.004*
RSES	24.06±5.43	16.07±7.78	p = 0.005*
PANSS_positive		13.23±2.39	
PANSS_negative		12.31±5.88	
PANSS_general		22.23±6.86	
PANSS_total		46.69±11.92	
Social distress (averaged score)	3.74±1.21	3.78±2.60	p = 0.752
Belonging	6.82±1.50	4.73±3.90	p = 0.149
Self-esteem	5.19±2.00	5.00±3.20	p = 0.922
Meaningful existence	1.17±1.96	1.74±2.96	p = 0.851
Control	1.78±1.51	3.17±3.61	p = 0.117
Manipulation check questionnaire	5.30±1.40	4.82±1.86	p = 0.839

Values are mean ± DS; NART, National Adult Reading Test; BDI, Beck depression inventory; SP, Spielberg anxiety scale; RSES, Rosenberg Self-Esteem Scale; PANSS, Positive and Negative Syndrome Scale; (*) significant difference between groups.

### Functional MRI data acquisition

For blood oxygen level dependent (BOLD) response imaging, T_2_* weighted gradient echo planar images were obtained using a GE Medical Systems Signa 1.5 T MRI scanner. A total of 30 axially orientated 5 mm thick contiguous sequential slices were obtained for each volume, 244 volumes being obtained with a TR of 2.5 s, TE 30 ms, flip 90°, FOV 240 mm and matrix 64×64. The first four volumes were discarded to allow for transient effects. A T_1_ weighted image was obtained to exclude gross structural brain abnormality.

### Social exclusion task

Subjects performed a version of the ‘Cyberball’ social exclusion task whilst being scanned [19]. In this task, subjects play a ball passing game with two cartoon animated figures on a screen, with the subject being represented by an animated hand (Fig. S1). Subjects were instructed to press either of two buttons to pass the ball to one of the cartoon figures. In turn, each cartoon figure either passed the ball to the subject or passed it to the other cartoon figure.

Throughout the task, the extent to which the subject was excluded in the game (ball not being passed to the participant) was systematically varied from 0% (ball equally shared between all three players) to 100% (ball only passed between the two animated figures). Specifically, the task was divided into 17 blocks with the following percentage levels of exclusion: 0, 25, 50, 75, 100, 75, 50, 25, 0, 25, 50, 75, 100, 75, 50, 25 and 0. As in previous studies, the behaviour of the two figures was driven by a computer program and the catching actions were performed automatically.

Participants had a short training session with Cyberball before playing the task in the scanner. They were instructed ‘when you receive the ball, just pass it back’. Subjects were not told that the object of the game was to study the effects of varying social exclusion. Participants were not told they were going to play with real people but were encouraged to ‘imagine the game as being with real people’. This was because a fully believable story about playing with others was impractical given the limited (controlled) behaviour of the cartoon figures. Also, previous research has shown that subjects experience similar level of distress when playing Cyberball against a computer as when they think they are playing against real people [20]. A very similar approach has recently been used in another Cyberball study [13]. To enhance the sense that the two cartoons represented real people taking decisions, the time that the cartoon figures took to pass the ball was randomly varied between 800 and 3000 milliseconds, simulating ‘decision making’. The task lasted for ∼10 minutes and was completed on a single run. All blocks lasted for the same length of time (∼35 sec) but varied slightly depending on the reaction time of the participant and the ‘decision making’ variation. There were no rest blocks.

### Clinical, behavioural and social ratings

Immediately before scanning, all subjects completed the Beck Depression Inventory (BDI) [21] Spielberger State Anxiety scale [22] and the Rosenberg Self-Esteem Scale [23]. Patients were additionally assessed using the Schizophrenia Positive and Negative Syndrome Scale (PANSS) [24]. Recruitment, clinical and rating scale assessment of all subjects, was by JDS, a Consultant Psychiatrist with considerable experience in the NHS.

After scanning, each subject was assessed using a self-report ‘social distress’ rating questionnaire [10] used in previous Cyberball studies [13]. This measure is predicated on the idea that ostracism threatens four primary social ‘needs’: belonging, self-esteem, control and meaningful existence [10]. Each need was assessed by a 0 to 10 point question, ranging from 0 (not at all) to 10 (very much). *Belonging* was assessed by the question “How much do you feel you belonged to the group?", *self-esteem* by the question “To what extent do you think the other participants value you as a person?", *meaningful existence* by the question “How true is the statement: ‘Life is meaningless’?" and *control* by the question “How true is the statement: ‘I am in control of my life’?". The questions were scored so that higher scores indicate a greater challenge to the social need. Additionally, participants completed a manipulation check similar to Williams and colleagues [10] that assessed mood, perceived intensity of ostracism and perception of group cohesiveness during the game. Social distress and emotional impact measures were analysed using multiple linear regression with group as a fixed factor and gender as a covariate.

### Image analysis

SPM8 (http://www.fil.ion.ucl.ac.uk/spm) was used for analysis. For pre-processing, global effects were removed from the fMRI time series using a voxel-level linear model of the global signal [25] (http://code.google.com/p/lmgs4spm). Images were slice-time corrected and realigned to the first image in each time series. The average realigned image was used to derive parameters for spatial normalization to the SPM8 Montreal Neurological Institute (MNI) template with the parameters applied to each image of the time-series. The resultant time-series realigned and spatially normalised images were finally smoothed with an 8 mm Gaussian kernel.

For first level analysis, a blocked design was implemented as a parametric modulation of percentage of social exclusion. This aimed to identify brain regions which activated as the degree of social exclusion systematically increased or decreased. Each subject's motor response times were included as a regressor to control for motor and associated cognitive processes during the task. The six head motion realignment terms where also included as further covariates of no interest, to allow for residual movement artefacts not removed by pre-processing realignment. The social exclusion and motor response regressors were convolved with the SPM8 haemodynamic response function without time or dispersion derivatives. For each subject, the covariate image used for second level analyses was the SPM8 ‘beta’ image, which comprised the estimated linear regression coefficient between the percentage of social exclusion and observed BOLD signal.

Two second level random effects analyses were conducted. The *first* consisted of testing the null hypothesis of no significant relationship between systematically increasing (or decreasing) exclusion and the observed brain response within each group (controls and schizophrenia). This was done by entering the covariate images of interest into two one-group t-tests. The *second* (second level) analyses consisted of testing the null hypothesis of no difference between control and patient groups in the imaging parameter estimates corresponding to the parametric regressor of social exclusion. The between groups comparison was performed using multiple linear regression with group as a covariate of interest and gender as a covariate of no interest. Both for the within and between group analyses, regions are reported that are significant at a cluster threshold of p<0.05 with whole brain correction. Monte Carlo simulations [26] indicated this was achieved by the *simultaneous* requirement for a voxel level threshold of p<0.005 and at least 106 continuous resampled voxels.

Next we investigated whether abnormal neural responses to social exclusion correlated with illness severity measures in the schizophrenia group. First, the positive and negative symptom scales of the PANSS were used in separate analyses as explanatory variables for a random effects whole brain regression analysis, of the parameter estimates for increasing social exclusion. For these regression maps we applied a cluster extent threshold of 141 voxels to ensure a *p*<0.05 threshold corrected for multiple comparisons across the whole brain with an individual voxel threshold of *p* = 0.05. Second, we examined whether significant activations in the regression maps overlapped with the mPFC region where patients differed from controls in their neural responses to social exclusion. In an analogous way, we tested for correlations with the self-report scores from the social distress and manipulation check questionnaires both for the control and schizophrenia group (results from this last analysis are reported in the supplementary material.

To examine whether neural response abnormalities in schizophrenia were secondary to antipsychotic medication we tested for correlations between brain activations in response to increasing degrees of exclusion and medication dose as chlorpromazine dose equivalents [27], [28] at a less stringent threshold of p<0.05 uncorrected.

## Results

### Clinical, behavioural and social ratings

Mean rating scale scores for each study group are shown in Table 1. As expected, between group t-tests identified significant group differences in mood as measured by the BDI (*t_(12.97)_* = 3.86, *p* = 0.002), Spielberger state anxiety (*t_(25)_* = 3.14, *p* = 0.004) and Rosenberg Self-esteem scale (*t_(27)_* = 3.02, *p* = 0.005), with patients rating themselves lower in mood and self-esteem and higher in anxiety than controls.

The number of button presses and mean reaction times during Cyberball were analysed using multiple linear regression with group as a fixed factor and gender as a covariate. No significant group differences were found. The social distress questionnaire aimed to assess the extent to which the Cyberball paradigm challenged participants needs (belonging, self-esteem, meaningful existence and control; see Table 1). There were no significant between group differences in individual need scores, or in the overall average social distress score (*F*_(1,26)_ = 0.102, *p* = 0.752). There were no between group differences in the ‘manipulation check’ (*F*_(1,26)_ = 0.042, *p* = 0.839) scores. This indicates patients were engaged with the task and perceived the varying inclusion/exclusion effect during Cyberball in a similar manner as controls. Consistent with the latter, all subjects indicated in informal discussions after scanning that they had noted being excluded during the game.

### Imaging Results

#### Within group analysis

**Figure 1:**
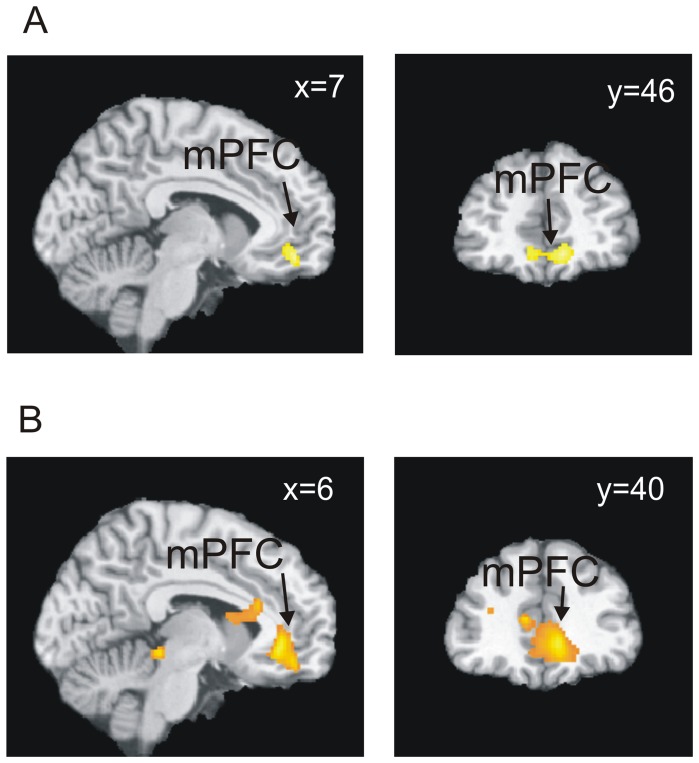
Analysis of neural responses to increasing social exclusion. (A) Neural responses to increasing social exclusion in the mPFC of controls. (B) Between group differences: controls exhibited greater strength in the relationship between increasing exclusion and brain activity in the mPFC than patients. All images are thresholded at p<0.05 corrected. Bottom right: plot of the parameter estimates for increasing social exclusion averaged across voxels in a 10 mm diameter sphere centred at (6,38,−4). Error bars represent 95% confidence intervals.

Replicating previous work, [13] controls exhibited a significant increase in the BOLD response with increasing social exclusion in a region extending through the mPFC/vACC and orbitofrontal cortex (OFC) (Fig. 1A). In contrast, patients did not exhibit significant activations with increasing degree of social exclusion. For the opposite parametric modulation (increased brain activation as social exclusion decreased) both groups exhibited significant clusters in several regions as detailed in Table 2.

**Table 2:** Within group brain activations as social exclusion increases or decreases.

Brain Region	BA	x	y	z	Z
*Activations with increasing social exclusion*					
Controls					
mPFC (medial frontal gyrus)	10	8	48	−12	3.39
*l* Occipital cortex (cuneus)	19	−14	−92	26	3.65
*r* Temporal cortex (superior temporal gyrus)	42	56	−30	16	3.50
Schizophrenia					
No significant activations					
*Activations with decreasing social exclusion*					
Controls					
*l* Dorsolateral frontal cortex (middle frontal gyrus)	6	−50	2	40	5.15
*l* Dorsolateral prefrontal cortex (middle frontal gyrus)	46	−42	40	32	3.88
*l* Superior parietal cortex	7	−26	−58	60	3.83
*r* Superior parietal cortex (precuneus)	7	10	−64	52	3.72
Schizophrenia					
*l* Dorsal anterior cingulate	32	−14	30	30	4.79
*l* Superior caudate	-	−14	0	20	4.12
Posterior brain stem	-	−2	−30	−6	4.65
*l* Inferior temporal cortex (fusiform gyrus)	20	−48	−6	−28	3.69
*r* Cerebellum	-	10	−78	−28	3.66
*r* Cerebellum	-	40	−62	−42	3.59
*l* Parietal cortex (precuneus)	7	−16	−58	58	3.49

Coordinates (x, y, z) reported in MNI space; mPFC, medial prefrontal cortex; *r*/*l* = right/left. All results significant at p<0.05 corrected. The Z value of the peak voxel of the region is reported.

#### Between group analyses

Group comparisons revealed significant differences between patients and controls in neural responses to increasing social exclusion (Table 3). Controls exhibited a greater increase in BOLD response with increasing social exclusion in the mPFC/vACC and OFC, compared to patients (Fig. 1B). This difference between patients and controls was also significant when only comparing the males of each group (see Fig. S2), showing that gender imbalance was not a cause of the results.

**Table 3:** Group comparison in the strength of the relationship between increasing social exclusion and brain activity.

Brain Region	BA	x	y	z	Z
Controls>Schizophrenia					
mPFC	10-11-24-32	6	38	−4	3.52
Superior caudate	-	−22	18	24	4.71
Posterior brain stem	-	0	−30	−8	4.57
Schizophrenia>Controls					
No significant activations					

Coordinates (x, y, z) reported in MNI space; mPFC, medial prefrontal cortex. All results significant at p<0.05 corrected. The Z value of the peak voxel of the region is reported.

It has been reported that self-esteem can modulate neural responses to social feedback in the mPFC [29]. Since the schizophrenia group showed significantly reduced self-esteem scores compared to controls, we tested whether between group differences in the mPFC would remain after controlling for self-esteem score group differences. Controls still demonstrated significantly stronger neural responses to increasing social exclusion in the mPFC compared to patients ((6, 38, −6), Z = 3.60, k_E_ = 1374, p<0.05 whole brain corrected). In a *post hoc* analysis, we performed a further analysis, controlling for self-esteem plus mood and anxiety ratings. This analysis showed that controls still exhibited stronger responses to social exclusion in the ventral and rostral mPFC than patients ((4,38,−6), Z = 3.13, k_E_ = 201, p<0.05 whole brain corrected). With the reverse test (schizophrenia patients exhibiting greater activation than controls as exclusion increased) no significant differences were found.

#### Correlations with symptom severity ratings in the schizophrenia group

**Figure 2:**
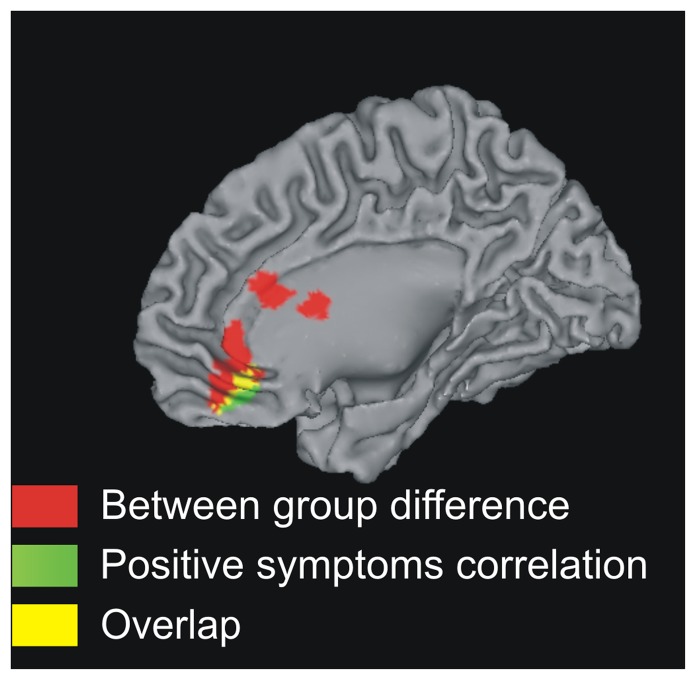
Correlation with positive symptoms in schizophrenia. Red: controls showed significantly stronger neural responses to increasing social exclusion than patients. Green: mPFC correlation between increasing exclusion and brain activity modulated by positive symptoms. Yellow: overlap between the Red and Green regions.

Our whole-brain regression analysis identified a region in the mPFC ((6,40,−14), Z = 2.41, k_E_ = 155 voxels, p<0.05 whole brain corrected) where the BOLD response to increasing social exclusion correlated negatively with scores from the PANSS positive symptom scale in schizophrenia. This cluster of activation overlapped with the mPFC foci where patients differentiated from controls in neural responses to social exclusion (Fig. 2). This indicates that reduced responses to increasing social exclusion were associated with increased severity of positive symptoms. In a *post hoc* qualitative analysis we explored which items of the PANSS positive symptom scale contributed to this correlation. This was done by examining whether the mPFC cluster in the regression map would increase or decrease in size (increased cluster size implies a better model of brain function), when the regression analysis was repeated, excluding one at a time each of the seven items of the PANSS positive symptom scale. Excluding symptoms of ‘delusions’, ‘grandiosity’ and ‘hallucinations’ reduced the mPFC cluster in the regression map, indicating these were contributors to the observed correlation. In contrast, excluding symptoms of ‘conceptual disorganization’ and ‘suspiciousness’ increased the cluster size, indicating these factors contributed variance and did not contribute to the effect. Excluding symptoms of ‘excitement’ and ‘hostility’ had no effect since these symptoms were absent in our group of patients.

No significant activations were found in the mPFC in regression analyses using the negative PANSS scale or the total PANSS score. Furthermore, no significant correlation activations were found with other non-core schizophrenia illness measures such as the BDI mood rating, Spielberg anxiety or self esteem Rosenberg scale ratings in the schizophrenia group. No correlation between the neural response to increasing social exclusion and antipsychotic dose (calculated as chlorpromazine equivalent dose) was found across the mPFC.

#### Correlations with self-report behavioural measures

In the control group, no significant correlations were observed in the mPFC between neural responses to social exclusion and ratings from the social distress and manipulation check questionnaires. In the schizophrenia group, a cluster was found in the mPFC were neural responses to increasing social exclusion correlated positively with scores from the social distress questionnaire ((0,38,−8), Z = 2.63, k_E_ = 386 voxels, p<0.05 whole brain corrected, Fig. S3). This cluster partially overlapped with the mPFC region where controls differentiated from patients. This means that stronger responses to social exclusion were associated with higher self-report measures of social distress in the schizophrenia group. Since both the positive symptom scores and the social distress scores correlated with neural responses in the mPFC we tested for a correlation between these two scales and no significant correlation was found (p = 0.86).

## Discussion

The aim of this study was to investigate hypothesised abnormalities in the neural correlates of social exclusion in schizophrenia. Replicating previous work, healthy controls responded to increasing exclusion by activating the mPFC/vACC and orbitofrontal cortex [13]. In contrast, patients with schizophrenia did not exhibit this response, with the magnitude of the abnormality correlating with positive symptom severity. Patients did not differ from controls in self-reported social distress measured immediately after scanning, consistent with a previous non-imaging study of schizophrenia [16]. This indicates that patients understood the task and perceived the inclusion/exclusion effect of Cyberball similarly to controls. The results are also broadly consistent with reports of mPFC abnormalities in schizophrenia during other social information processing tasks [7].

Consistent with our findings in controls, activation of the mPFC/vACC in response to social exlusion has been reported in other Cyberball studies [13]–[15]. The ventral and rostral mPFC activates during self-evaluation and mentalising tasks that require inferences about other people's thoughts [8], [30]–[33]. Social exclusion may trigger processes of self-evaluation [34] and reflections on the mental states of others, both being linked to increased activity of the mPFC [13]. Failure to activate the mPFC with increasing exclusion in schizophrenia suggests the degree of exclusion did not modulate processes of self-evaluation or generation of inferences about other's beliefs.

The ventral mPFC has been implicated in representing/updating the expected *value* of reward and punishment outcomes and using this information to guide behaviour [35]–[37]. This role may extend to monitoring of social exchange outcomes [8], [38]. As increasing social exclusion may result in closer monitoring and updating of social values to plan future behaviour [13], our findings suggest dysfunction of social valuation processes in schizophrenia.

In schizophrenia, a reduced mPFC response to social exclusion correlated with increased severity of positive symptoms. This is consistent with patients exhibiting different levels of social performance at different periods of illness, with remitted patients performing better that patients during an acute phase of illness [39]. This observation can be interpreted in the light of an influential theory [40] that postulates an inappropriate attribution of motivational significance to external and internal stimuli driving psychotic symptoms. The theory argues that delusions arise as an attempt to make sense of the experience of aberrant salience, with hallucinations arising more directly due to the aberrant salience of internal percepts and memories. Supporting this, fMRI studies have reported that schizophrenia patients fail to make a distinction at a neural level, between normally salient (rewarding or aversive) and non-salient (neutral) events, with the magnitude of this abnormality correlating with delusional severity [41], [42]. Similarly, patients in our study failed to alter mPFC activation with percentage of exclusion, with this abnormality correlating with positive symptom severity. Abnormal attribution of salience has been linked to a disturbance in dopamine signalling [40] and an important target of dopamine neurons is the mPFC [43], [44]. Our finding of abnormal mPFC responses to increasing social exclusion could therefore reflect a failure of dopamine firing to assign normal salience to social feedback, but further work is required to test this hypothesis. Interestingly, it has been proposed that a disturbance in mPFC dopamine signalling underlies abnormalities observed in social cognition studies of schizophrenia [7].

While mPFC activation in controls replicates previous work, [13]–[15], we did not observe other findings reported in some Cyberball studies, such as increased activation during exclusion versus inclusion in the dorsal anterior cingulate, ventrolateral prefrontal cortex, insula and amygdala [11], [12], [45]. These differences may be due to different methodological approaches. For example, we used parametric modulation of the degree of exclusion, with previous studies using single inclusion and exclusion blocks [11], [12] or multiple randomised exclusion/inclusion blocks [13]. In addition, we covaried out motor response times to control for motor and motor-associated cognitive effects.

While no correlation was observed in controls between self-reported distress and BOLD responses in the mPFC, a significant correlation was found in the schizophrenia group. In patients, stronger neural mPFC responses to social exclusion were associated with higher levels of self-reported social distress. This finding in schizophrenia is consistent with a previous study reporting a correlation between self-reported distress and neural activation during exclusion versus to inclusion, in the subgenual anterior cingulate cortex [45]. The wider range of social distress scores in schizophrenia may have increased the power to detect a relationship.

Potential limitations should be noted. The sample size was limited although the numbers of subjects are reasonably typical for a clinical imaging study. Thus, it is important to replicate findings using larger samples. The schizophrenia group had a higher male to female ratio than the control group, therefore gender was used as a covariate in the analysis. In addition, the analysis was repeated with only male subjects, replicating the findings. This indicates that gender was not a confound, but the results are particularly relevant for males with schizophrenia. Patients were receiving antipsychotic medication at the time of the study. However, no correlations were observed between chlorpromazine equivalent doses and brain activity, but correlations between illness severity and brain activity were present, suggesting the results were not secondary to antipsychotic medication. While the image analysis demonstrated differences between patients and controls in neural responses to social exclusion, the behavioural analysis did not show differences in self-report measures of social distress. Whilst it is reassuring that patients reported noticing exclusion during Cyberball similarly to controls (this indicates that patients were engaged with the task and that neural differences are not likely a consequence of simple inattention or lack of understanding) it would be worthwhile trying to develop additional self-report measures which are sensitive to differences between groups. Finally, it would be interesting to perform similar studies with other psychiatric populations to test how specific our findings are to schizophrenia.

To our knowledge, this is the first study to investigate the neural substrates of social exclusion in schizophrenia. Compared to controls, patients with schizophrenia failed to modulate activity in the mPFC/vACC and orbitofrontal cortex, in response to varying degrees of social exclusion. This may reflect altered modulation of social information processing in response to social feedback, highlighting the mPFC as a potential neural substrate of interpersonal difficulties in schizophrenia.

## Supporting Information

Figure S1 **The Cyberball paradigm.** The ‘hand’ at the bottom represents the real subject's actions.
(TIF)

Figure S2 **Between groups analysis of neural responses to social exclusion including only male participants.** Controls exhibited greater strength of relationship between increasing social exclusion and neural activity in the ventral and rostral mPFC compared to patients. Image region significant at p<0.05 corrected (see Methods section for details).
(TIF)

Figure S3 **Correlation with self-reported social distress in schizophrenia.** Red: regions where controls showed significantly stronger neural responses to increasing social exclusion than patients. Green: region in the mPFC where the strength of the correlation between increasing exclusion and brain activity correlated positively with self-reported social distress in schizophrenia. Yellow: overlap between the significant between groups and correlation regions.
(TIF)

Table S1 Patients antipsychotic medication and chlorpromazine equivalents.
(DOC)
