# Supplementary figures and images for: Abnormal Neural Responses to Social Exclusion in Schizophrenia
Source: PLoS One. 2012 Aug 16;7(8):e42608. doi: 10.1371/journal.pone.0042608 (PMC3420898; doi:10.1371/journal.pone.0042608)

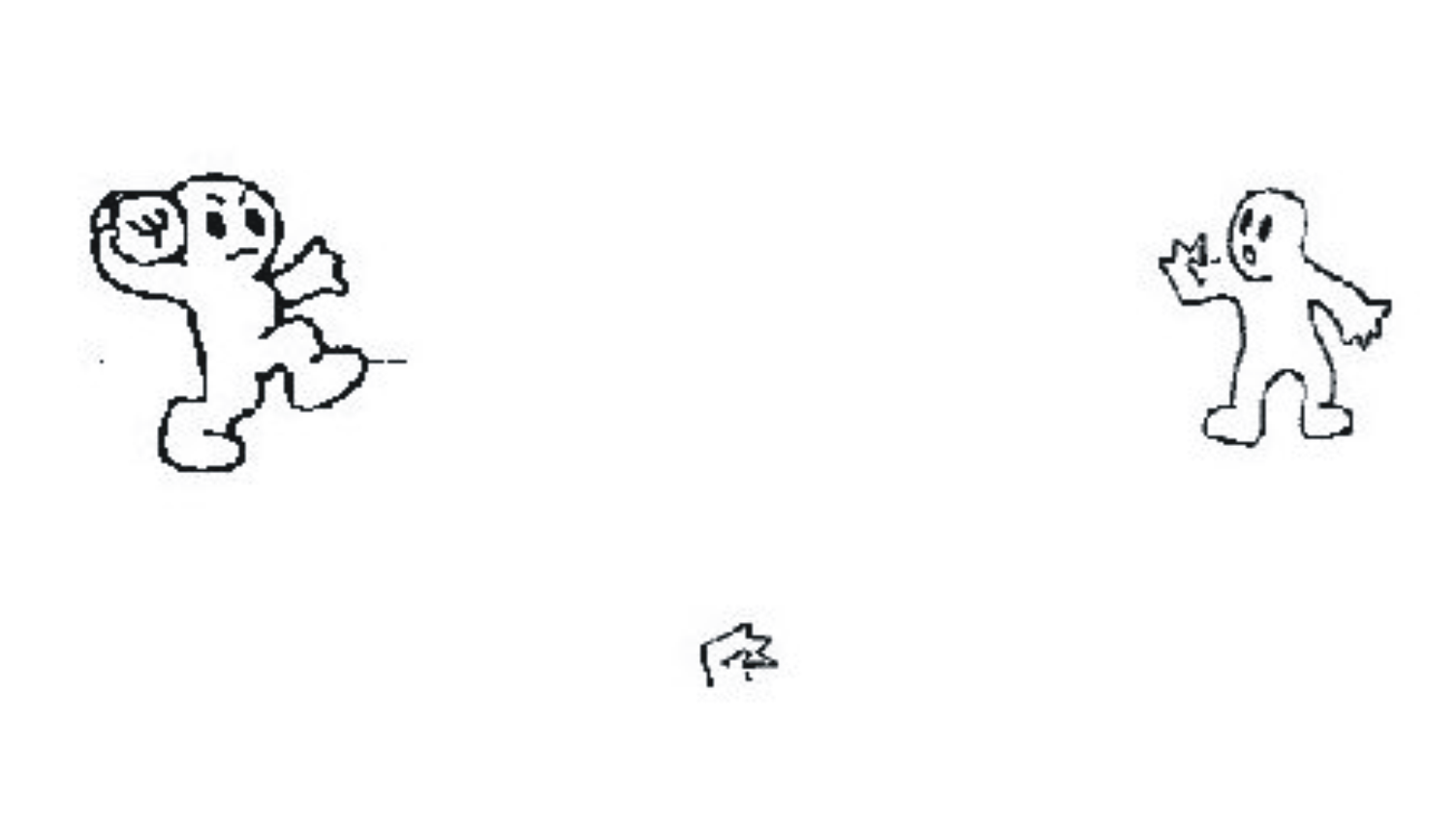

Supplement: Figure S1 — The Cyberball paradigm. The ‘hand’ at the bottom represents the real subject's actions. (TIF) [file pone.0042608.s001.tif]

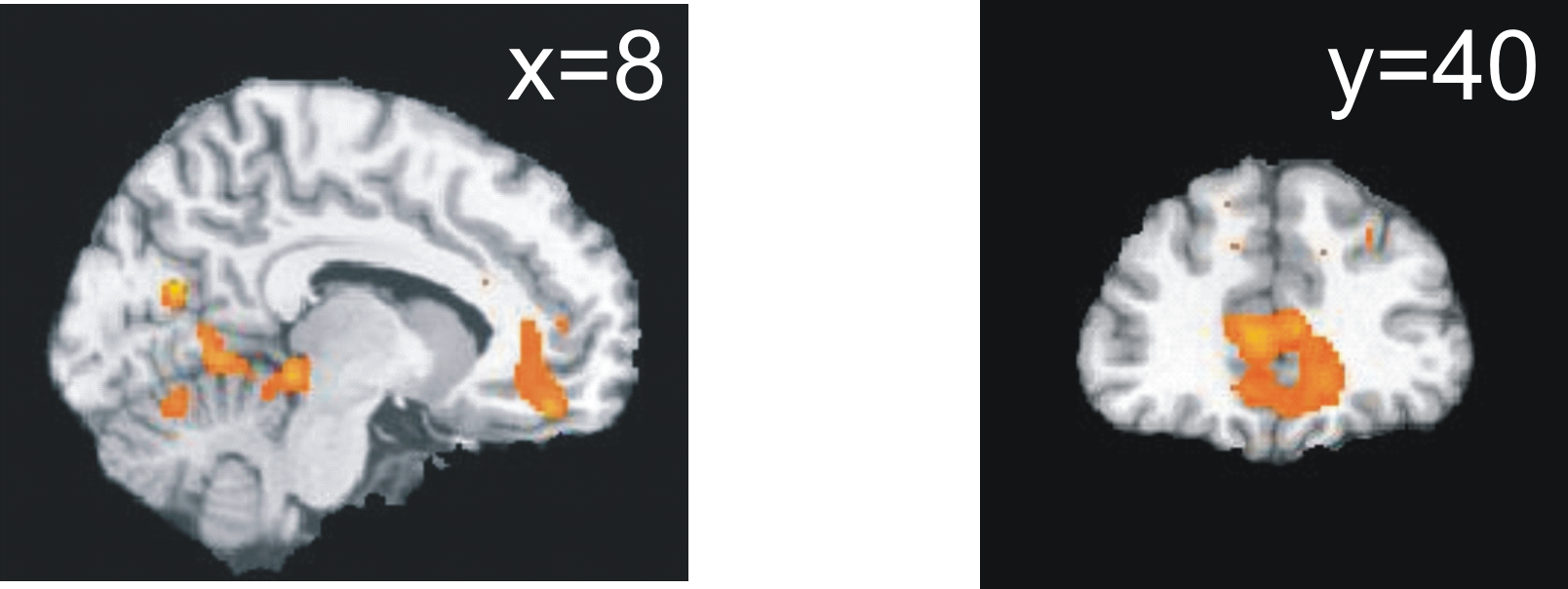

Supplement: Figure S2 — Between groups analysis of neural responses to social exclusion including only male participants. Controls exhibited greater strength of relationship between increasing social exclusion and neural activity in the ventral and rostral mPFC compared to patients. Image region significant at p<0.05 corrected (see Methods section for details). (TIF) [file pone.0042608.s002.tif]

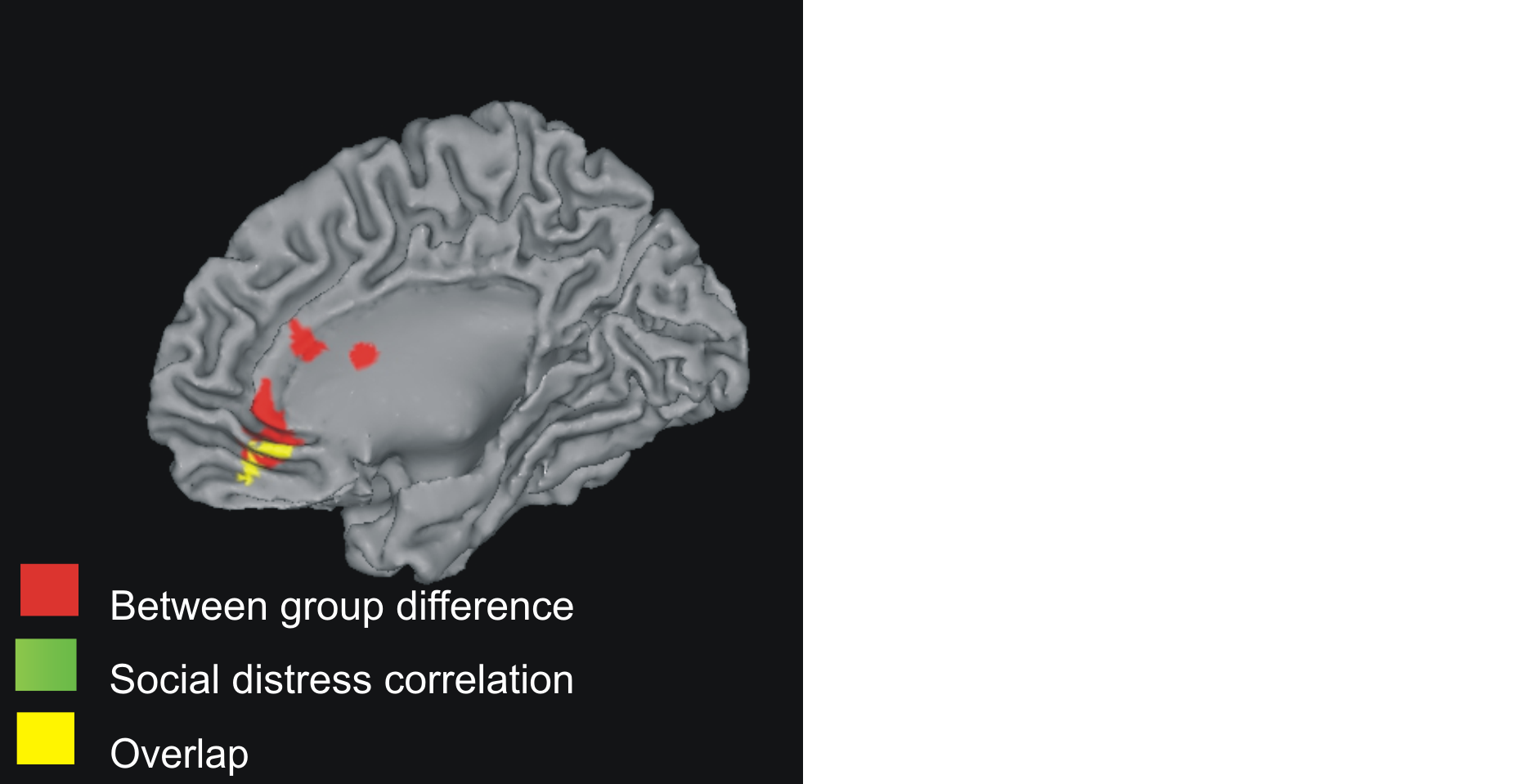

Supplement: Figure S3 — Correlation with self-reported social distress in schizophrenia. Red: regions where controls showed significantly stronger neural responses to increasing social exclusion than patients. Green: region in the mPFC where the strength of the correlation between increasing exclusion and brain activity correlated positively with self-reported social distress in schizophrenia. Yellow: overlap between the significant between groups and correlation regions. (TIF) [file pone.0042608.s003.tif]
