# Supplementary material for: Abnormal Neural Responses to Social Exclusion in Schizophrenia
Source: PLoS One. 2012 Aug 16;7(8):e42608. doi: 10.1371/journal.pone.0042608 (PMC3420898; doi:10.1371/journal.pone.0042608)
Supplement: Table S1 — Patients antipsychotic medication and chlorpromazine equivalents. (DOC) [file pone.0042608.s004.doc]

**Supplemental Material**

**Supplementary Table S1**

Patients antipsychotic medication and chlorpromazine equivalents.

| **Antipsychotic medication** | **Chlorpromazine equivalents (mg/day)** |
| --- | --- |
| Quetiapine 700 mg daily | 933 |
| Pipothiazine palmitate 50 mg every 4 weeks | 125 |
| Clozapine 900 mg daily | 900 |
| Flupenthixol Decanoate 200 mg depot every 3 weeks | 667 |
| Clozapine 500 mg daily | 500 |
| Risperidone 3 mg twice a day | 400 |
| Olanzapine 10 mg twice a day | 400 |
| Olanzapine 20 mg daily | 400 |
| Clozapine 250 mg daily | 250 |
| Quetiapine 600 mg daily | 800 |
| Clozapine 325 mg daily | 325 |
| Clozapine 350 mg daily | 350 |
| Quetiapine 250 mg daily | 333 |
